# Fe-doped mayenite electride composite with 2D reduced Graphene Oxide: As a non-platinum based, highly durable electrocatalyst for Oxygen Reduction Reaction

**DOI:** 10.1038/s41598-019-55207-6

**Published:** 2019-12-24

**Authors:** Karim Khan, Ayesha Khan Tareen, Muhammad Aslam, Sayed Ali Khan, Qasim khan, Qudrat Ullah Khan, Muhammad Saeed, Awais Siddique Saleemi, Maryam Kiani, Zhengbiao Ouyang, Han Zhang, Zhongyi Guo

**Affiliations:** 1 Advanced electromagnetic function laboratory, Dongguan university of Technology, Dongguan, Guangdong, China; 2 Shenzhen Engineering Laboratory of Phosphorene and Optoelectronics, and SZU‐NUS Collaborative Innovation Center for Optoelectronic Science and Technology, Shenzhen University, Shenzhen, 518060 China; 3 College of Electronic Science and Technology, Shenzhen University, THz Technical Research Center and Key Laboratory of Optoelectronics Devices and Systems of Ministry of Education and Guangdong Province Shenzhen University, Shenzhen, 518060 China; 4 Government Degree college PaharPur, Gomel University, Dera Ismail Khan, K.P.K., Islamic Republic of Pakistan; 5 Institute for Advanced Study, Shenzhen University, Shenzhen, Guangdong, 518060 China; 6 Key Laboratory of Optoelectronic Devices and Systems of Ministry of Education and Guangdong Provence, College of Optoelectronic Engineering, Shenzhen University, Shenzhen, Guangdong, China 518060; 7 College of Physics and Optoelectronics Engineering, Shenzhen University, Nanhai Ave. 3688, Shenzhen, Guangdong, 518060 China

**Keywords:** Chemistry, Materials science, Nanoscience and technology

## Abstract

Since the last decades, non-precious metal catalysts (NPMC), especially iron based electrocatalysts show sufficient activity, potentially applicant in oxygen reduction reaction (ORR), however they only withstand considerable current densities at low operating potentials. On the other hand iron based electrocatalysts are not stable at elevated cathode potentials, which is essential for high energy competence, and its remains difficult to deal. Therefore, via this research a simple approach is demonstrated that allows synthesis of nanosize Fe-doped mayenite electride, [Ca_24_Al_28_O_64_]^4+^·(e^−^)_4_ (can also write as, C_12_A_7−x_Fe_x_:e^−^, where doping level, x = 1) (thereafter, Fe-doped C12A7:e^−^), consist of abundantly available elements with gram level powder material production, based on simple citrate sol-gel method. The maximum achieved conductivity of this first time synthesized Fe-doped C12A7:e^−^ composite materials was 249 S/cm. Consequently, Fe-doped C12A7:e^−^ composite is cost-effective, more active and highly durable precious-metal free electrocatalyst, with 1.03 V onset potential, 0.89 V (RHE) half-wave potential, and ~5.9 mA/cm^2^ current density, which is higher than benchmark 20% Pt/C (5.65 mA/cm^2^, and 0.84 V). The Fe-doped C12A7:e^−^ has also higher selectivity for desired 4e^−^ pathway, and more stable than 20 wt% Pt/C electrode with higher immunity towards methanol poisoning. Fe-doped C12A7:e^−^ loses was almost zero of its original activity after passing 11 h compared to the absence of methanol case, indicates that to introduce methanol has almost negligible consequence for ORR performance, which makes it highly desirable, precious-metal free electrocatalyst in ORR. This is primarily described due to coexistence of Fe-doped C12A7:e^−^ related active sites with reduced graphene oxide (rGO) with pyridinic-nitrogen, and their strong coupling consequence along their porous morphology textures. These textures assist rapid diffusion of molecules to catalyst active sites quickly. In real system maximum power densities reached to 243 and 275 mW/cm^2^ for Pt/C and Fe-doped C12A7:e^−^ composite, respectively.

## Introduction

Clean and renewable energy production is important concern that can replace the conventional energy production devices. Conventional energy producing devices work on depletion of fossil fuels i.e. coal, methane gas, petroleum, etc, which are one of the greatest challenges of this era due to lethal environmental impacts. Increasing pollution anxiety coupled with fossil fuels utility, calling another more sustainable, substituent energy source^1–4^. Thus, exploring a cleaner energy production and saving device like fuel cells, metal-air batteries etc, with more suitable catalysts strategy is urgently needed for automotive, residential and portable electronic applications. Hence, to meet future energy requirements regarding to renewable energy production, hydrogen (H_2_) based fuel cells are possibly one of future alternatives for conventional fossil fuels based devices. The innovation in fuel cell based technology might be most capable promise for electric automobiles. Hydrogen based fuel energy production is extensively pursued because, it is potential competitor owing to invincible energy density with approximately not releasing green house gases. The H_2_ is economically abundant fuel, which help us in getting electrical energy from chemical energy under redox exothermal reaction. Despite great progress in past decades, large scale materialization of the fuel cell technology is restricted to some extents because of their sluggish kinetics of the ORR with their high cost due to use of a massive quantity of less durability Pt-based electrocatalysts. Presently, the scheme design for synthesis of high-performance, durable, low-cost ORR electrocatalysts in fuel cell is still a significant challenge.

Choice of catalyst is critical as it can directly affect the efficiency, durability, and the cost of the fuel cell. Therefore, developing highly efficient low cost electrocatalysts for ORR is a key to fabricate long time commercially viable fuel cell device for future energy applications. The established fuel cell technology is entirely based on Pt group metals (PGM) electrocatalysts, which cost almost 40 to 60% of fuel cell heap. It is predicted that electrocatalysts inflation will further goes on with the further industrialization of renewable energy devices due to scarcity of Pt based electrocatalysts. This is alarming situation by using the PGM-based electrocatalysts, so motivated the researchers toward NPMCs group. So, lots of struggles are continuing to expand research interests in materials chemistry for electrocatalysts that can endorse the ORR, thus improving fuel cells performance. Even though noble metal (NM) based electrocatalysts, e.g. Pt alloys, are established to show catalytic properties in ORR however, they still now suffering from a number of the serious obstacles, counting electrode reduced steadiness in fuel cell surroundings, and the diminutive tolerance towards poisoning. Therefore, we need to make strategy to overcome these challenges by developing low or free of PGMs based catalysts, to lower the cost but maintain or even improve the ORR activity.

To get rid of the PGM elements and lower the cost, many researchers have tried to exploit a low cost catalysts but still with some limitation^1^. Efficient precious-metal free catalysts for ORR are highly desirable in upcoming cost effective energy devices. Therefore, it is necessary to build up the low-cost, plentiful, energetic, and robust ORR electrocatalyst for alkaline medium. Porous, NPMCs with nanosize are more economical electrocatalysts for ORR, because these kinds of materials provide superior interface surface for O_2_ and vigorous catalyst sites. Similarly, mixing different vigorous elements may strengthen or alter each other to enhance the ORR performance using possible synergistic effects. The strategies to design hybrid structures to advance the ORR catalysts fabrication is also very important but remains a huge confront in uniform synthesis of one ORR active material on other material to perform their best.

Since the last decade, the NPMCs using cobalt or iron like metal dynamic midpoint demonstrate enough activity that is fruitful for ORR. These metals cations can maintain considerable current densities as redox reactions occurred at small onset potentials, wherever the cathode is restricted with mass-transfer and kinetics; final polarization graph have repeatedly exposed a subordinate ORR property. Although, for high energy efficiency, their stabilization at higher potentials is necessary to maintain intimidating task due to their less stability. Therefore, poor stability of previously established NPMCs in fuel cell operation was foremost dilemmas hamper their industrial claim to make them more economical. The 2D materials also plays very important role in this direction. Hence, via this research work we focused on stabilizing iron-carbon (Fe-C) based like catalyst over whole potential range through doping “Fe” in the C12A7:e^−^ to further enhance the electrical properties and coating carbon as rGO to make it more stable, which would make it viable for the fuel cell^1,5^. The C12A7:e^−^ is abundant main group elements, with natural nanocage structure, shows multiple functions due to theoretical support based experimental study for incorporation of cations doping that can enhance the clattered electron inside its sub-nanometer size cages. There are very few reports that shown the doping effect on morphology and crystallinity of versatile, ubiquitous C12A7:e^−^ electride material^1,5–12^. Regarding to the Fe doping, here we are the first to study its electrocatalyst properties. This electrocatalysts is earth abundant, cheaper, and own very rich redox properties, and also less toxic than other transition metals. Hence, these advantages inspired us to investigate the “Fe” doping in the C12A7:e^−^ electride as a electrocatalyst for the ORR in the fuel cell. Therefore, we schemed a technique to synthesize a suitable “Fe” doping in the C12A7:e^−^ electride, by partially replacing the “Al” with the “Fe” and also removal of free oxygen from the cages, based conduction for mass production that will acts as a more suitable, highly active, and stable electrocatalyst in ORR^13^. As a result it will acts as a low cost, durable electrocatalyst for fuel cell at commercial level^1,5–10^. Through in this research work we will discuss in detail the synthesis method, characterizations required to analyze it and finally its electrocatalytic applications in fuel cell.

## Fe-doped C12A7:e^−^ Composite Synthesis

In this section, we are going to scheme and applied a general and efficient strategy to synthesize Fe-doped C12A7:e^−^ with rGO composite nanomaterials that will evaluate its ORR activity. In a typical synthesis approach “Fe” doping in C12A7:e^−^, carried out with enhanced electrical properties, that are very helpful for electrocatalytic ORR properties, that will commercialize the fuel cell technology under economical circumstances, for future pollution free energy devices^14^.

### Chemicals

The Ca(NO_3_)_2_·4H_2_O, Al(NO_3_)_3_·9H_2_O, Fe_2_O_3_, Ethylene Glycol (EG) and Citric Acid (CA) all precursors were bought from Aladdin Bio-Chem Technology Co., Ltd (Shanghai China). The chemicals were used as such because they are analytical grade.

### Experimental scheme and synthesis

**Figure 1:**
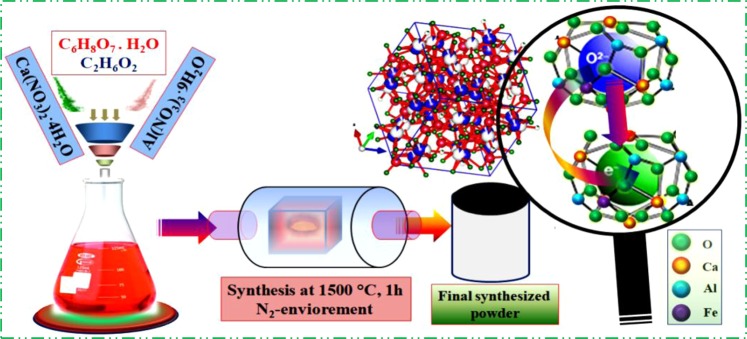
Synthesis scheme of Fe-doped C12A7:e^−^ electride.

The synthesis scheme that we are going to apply for production of the enhanced electrical conductive Fe-doped C12A7:e^−^ electrocatalyst composite is shown in Fig. 1. In typical synthesis method, first the stichometric ratios (12:14) of Al(NO_3_)_3_·9H_2_O and Ca(NO_3_)_2_·4H_2_O were weighted and Fe_2_O_3_ precursors for “Fe” doping with doping quantity, x = 1, was added simultaneously in the ethylene glycol and citric acid solution, at ~80 °C and stirred to get a transparent gel^1,5,7^. The ratio of CA to metal cations, CA/M_Ca,Al_ = 1/2.

To get viscous gel we further heated precursor’s solution at 130 °C for one hour and then slowly heat treated at 290 °C for 4 hours to get dried/xerox gel. Then it was crushed and reheated at 550 °C/1 h in N_2_ with heating rate 5 °C/min. The final product was again crushed, and half of sample was pressed (~150 MPa) for some measurements, especially the electrical properties measurement. The resultant powder and pellet were sintered at 1500 °C/1 h at same time in alumina crucible under N_2_ flow with thermal treatment and cooling rate of 4 °C/min.

Morphology, structure, elemental composition and porosity were comprehensively studied with different characterization techniques. First of all, the chemical reactions occur during sintering were determined using thermal gravimetric and differential thermal analyses (TG/DTA) (Diamond TG-DTA; Perkin-Elmer instrument (SII) Thermoplus, Rigaku). In next stage, temperature effect for required synthesized phase formation and its crystallinity were examined via X-ray powder diffraction (XRD) technique, using D8 Advanced Bruker AXS diffractometer via Cu-Kα radiation source (λ = 0.15406 nm, 40 mA, 40 kV). Raman spectroscopy was carried out to test carbon family in synthesized material. The Raman spectra excitation was obtained by means of a 532 nm argon ion laser (20 mW). Another important investigation in this research was to evaluate the doping effects on the electrical properties; therefore electrical conductivity of the sample was measured by four probe method. Contacts were improved by Pt-paste and conductivity and measured with in variable temperatures range of 90–500 K. X-ray photoelectron spectrometer (Thermo Scientific, VG Multilab 2000) was used for elemental and their phases evaluation by X-ray photoelectron spectroscopy (XPS) with Al-Kα radiation of energy 1486.6 eV, and under ultra-high vacuum (UHV) of 1.33 × 10^−8^ Pa. For wide and narrow scans, the applied energy resolutions were 0.5 eV and 0.05 eV, the analyzer surpass energy was 20 eV for narrow scans and peaks, and calibration was done by carbon C1s (284.8 eV). Finally, for microstructural analysis the final yielded material was investigated using the Scanning Electron Microscopy (SEM) and the Transmission Electron Microscopy (TEM) (JEOL-2100, 200 kV).

Regarding to the application point of view, electrocatalytic properties were also studied. The electrocatalytic properties of catalysts were calculated using cyclic voltammetry (CV) and rotating disk electrode (RDE) method on the MSR electrode rotator (Pine Instrument Co.), connected via computer controlled potentiostat, electrochemical CHI 760 C analyzer, consist of the three electrodes, which are saturated reference electrodes (Ag*/*AgCl/KCl), working electrode i.e. glassy carbon (GC) electrode, and a graphitic rod based counter electrode. To check ORR activity by electrode preparation, first of all ink was prepared by dissolving synthesized catalysts powder in isopropyl alcohol water, and nafion with volume ratios, 1:9:0.1, under sonication at room temperature (RT). Very little amount of nafion was helpful to make smooth dispersion of catalyst ink. For activity measurement, about 8 μl of ink casted on GC working electrode surface (diameter = 0.196 cm^−2^) and air dried at RT. Catalyst loading quantity of the active Fe-doped C12A7:e^−^ composite on working GC-electrode was ~0.1 mg.cm^−2^. The electrochemical activity was measured via CV analysis. Before measuring CV the electrolyte was 20 minute purged under O_2_ flow, at RT, in 0.1 M KOH. The CV cycle potential measured with 100 mV·s^−1^ rate in 0.05–1.2 V range, until stable CV curves were measured. Background currents were recorded in 1.2–0.2 V potential range under N_2_-saturated environment. Finally, linear sweep voltammograms (LSV) under O_2_-flow were recorded setting different rotations per minute (rpm) and evaluate the difference between the synthesized powders LSV obtained at 1600 rpm with standard 20% Pt/C. For alkaline ORR measurements, loading amount of Pt/C (20%) was ~0.1 mg.cm^−2^, which contain loaded Pt quantity of ~20 μg cm^−2^. Koutecky-Levich (K-L) relation was used to calculate the kinetic Parameters^15,16^. The mechanism of the mesoporous Fe-doped C12A7:e^−^ composite will be discussed in detail to practically demonstrate that the Fe-doped C12A7:e^−^ composite can exhibit higher electrocatalytic activity towards ORR in the fuel cell. The electrocatalyst activity in terms of their half wave potential, current density, and robustness results will be discuss in details.

### Fuel cell test

The synthesized electrocatalyst was used as a fuel cell cathode, and its performance in the real system was assessed by membrane electrode assembly (MEA) analysis in the AAEMFC. For the preparation of the cathode, the catalyst and commercial ionomer (50 wt%) were well dispersed in isopropyl alcohol (IPA) via sonication. Subsequently, the homogeneous ink of catalyst (0.1 mg cm^−2^) as a cathode and Pt/C (0.1 mg cm^−2^, which contain loaded quantity of Pt is ~20 μg cm^−2^) as an anode were loaded on the active area of the gas diffusion layer (GDL). The MEA was fabricated by sandwiching KOH-doped Tokuyama membrane between the cathode and anode. Finally, the MEA was conducted in a single cell mode, which comprises serpentine flow field channels in the graphite plates. The steady‐state polarization experiment (cell voltage and power) of the assembled MEA was measured at 80 °C by keeping a humidified flow (100% relative humidity) of the hydrogen and oxygen at the flow rate of 200 cc min^−1^^16^.

## Results and Discussion

### TG/DTA analysis

**Figure 2:**
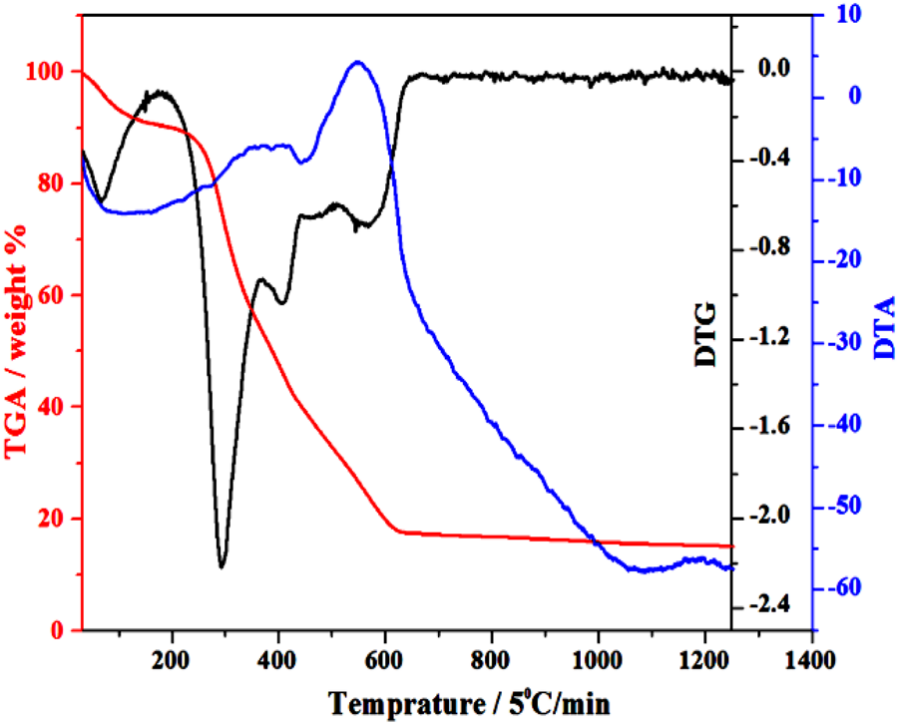
TG/DTA of precursor’s sample.

The basic compositional changes in the precursors during formation of the final material with required properties as a function of increasing temperature at a constant rate were studied by using the TG/DTA technique. The endothermic/exothermic reactions and phase transformation during the heat treatment were collected in the N_2_ environment, from 30 °C to 1250 °C (Fig. 2). From 100–150 °C, weight loss were observed by physically absorb water and dehydration reaction of crystal water in the nitrates evaporates^17^, and small endothermic peak confirmed the first citric acid decomposition below the 200 °C, where all citrate conversion after decomposition did not occurs into gaseous products due to the decomposition of nitrate under inert gas environment^18^. The brownish color of gel also confirmed the contamination of precursors by high amount of the unburned carbon^1,5,7^. The second obvious continuously weight loss around 250 °C to 700 °C with exothermal peaks, possibly due to the evaporation/decomposition/burning of the loosely bonded organic species like nitrogen^19^. Endothermic peaks at about 600 °C suggest complete decomposition of all meta-stable phases, which have stronger bonding with the metal species, therefore, they could decomposed at this temperature and also starting formation of the crystalline phase of the mayenite electride^20–22^. However, no distinct endothermic/exothermic peaks were observed at higher temperature range because of either C12A7:e^−^ oxidation of all free carbon.

At 600 °C the discrete exothermic peak is very important that shows oxidization of the free extra carbon from un-burnt carbon, which source is citric acid^19^. That oxidization of the free carbon maybe because of free oxygen reduction in the cages and hence will cause introduction of electrons in the samples, at temperature higher than^5^ 600 °C but at ≥ 700 °C has a discrete value of conductivity^5–8^. Based on TG/DTA and our previous experience, we selected 1500 °C as a final synthesis temperature, because it will also stabilize the synthesized material and hence will enhance the electrochemical properties for the ORR in fuel cell^1,5–10^. For further clarification of required phase(s), we did XRD of the as synthesized sample.

### XRD of Fe-doped C12A7:e^−^ composites

**Figure 3:**
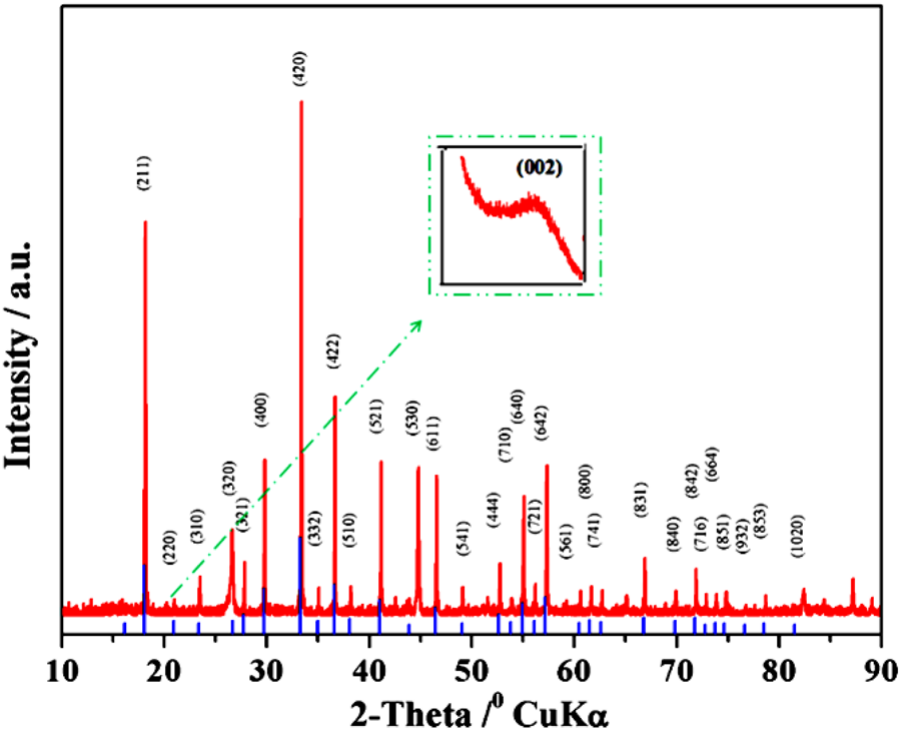
XRD patterns of C_12_A_7−x_Fe_x_:e^−^ samples (x = 1).

High temperature pyrolysis can extensively improve the crystallinity and hence the activity and stability of the Fe-doped C12A:e^−^ electrocatalysts^23,24^. In this research work, the XRD of the synthesized powder was carried out to analyze the phase **(**Fig. 3**)**. XRD based phase identification and crystallinity of Fe-doped C12A7:e^−^ showed majority peaks well agree with crystalline C12A7 phase (**JCPDS, CAS #48-1882**^5–8^.

Inset Fig. 3 shows graphitic carbon peak. Similarly, the doping doesn’t change the pristine structure of the C12A7 lattice framework, under the elevated heat treated. Hence, it shows that the applied controlled synthesis conditions are appropriate for the production of the Fe-doped C12A7:e^−^ but under previously introduced synthesis method for doped C12A7:e^−^ phase was unstable^25^. This unstability was due to reduction. The electrons generated as a result of reduction in mayenite cages and they undergoes into decay process, except the method introduced by our group removes these barriers^1,5^. The graphitic carbon peak was further confirmed by Raman spectroscopy.

### Raman spectroscopy

**Figure 4:**
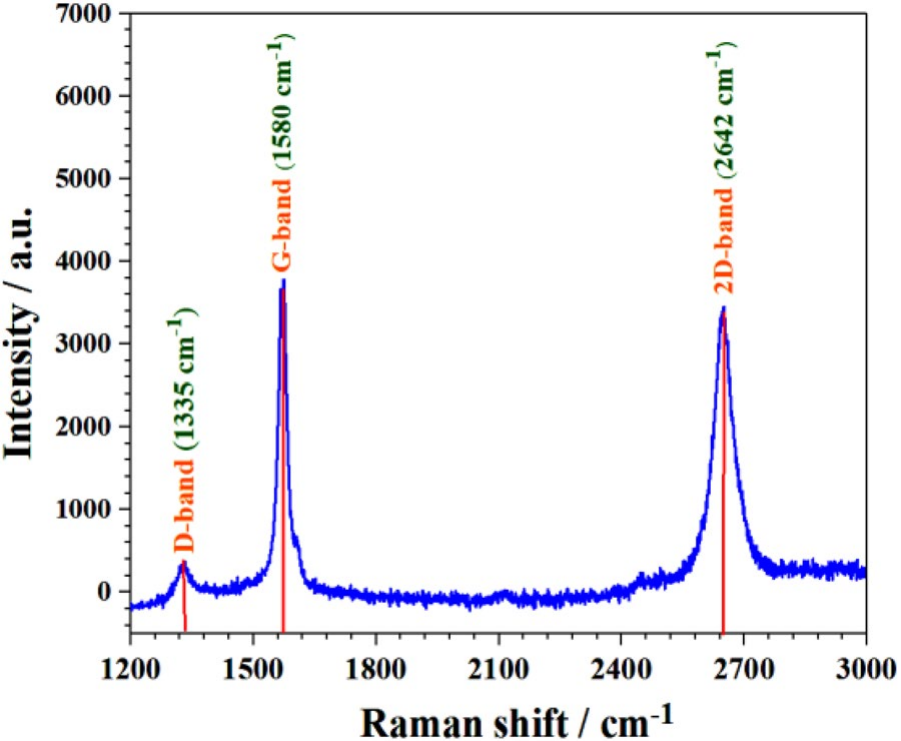
RAMAN spectra of C_12_A_7−x_Fe_x_:e^−^ samples, where doping level, x = 1.

Historical importance of the Raman spectroscopy is to investigate and characterize the bonding in the graphitic structure. Based on our previous experience, using ethylene glycole and citric acid work as a rGO source^5^. So, to further confirm it, Raman spectroscopy was carried out. The obtained Raman spectra of highly conductive Fe-doped C12A7:e^−^ composite was excited with a laser has 532 nm wavelength (Fig. 4). The highest intense G-band peak at ~1580 cm^−1^, shows sp^2^-hybridized carbon atoms, second intense 2D band peak was observed at 2642 cm^-1^, and third D-band was observed at about 1355 cm^−1^. Here, the I_D_/I_G_ ratio was about 0.2, indicating some impurities and defects in graphitized carbon structure. Raman spectroscopy confirm the rGO in the final sample, as we previously observed^1,5,7^. For further evaluation, SEM/TEM and XPS were studied.

### Microstructural analysis

**Figure 5:**
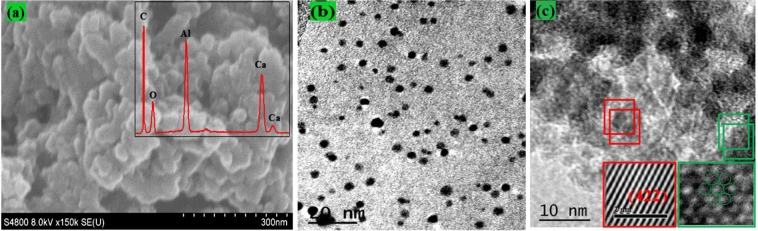
(**a**), SEM, and (**b**), TEM/HR-TEM images of C_12_A_7−x_Fe_x_:e^−^, where doping level, x = 1.

In case of the developing superior ORR catalysts, enhanced catalytic performance can also be accomplished by manipulating atomic structure, as well as surface electronic properties of catalysts, that is confirmed to be the most important proposed option. Therefore, we fabricated a rGO layered on the surface of Fe-doped C12A7:e^−^. The most important effect of the existence of rGO in material during synthesis is not only important for keeping long time electrical stability of the doped C12A7:e^−5^ but can also play a great role in synthesis of doped C12A7:e^−^ powder in nanosize by not allowing particles agglomeration at such high synthesis temperature. Morphological design of catalysts has one of great important catalytic activities which can effectively enhanced by synthesizing nanosize particles and hence improving mass transport. The Fe doped C12A7:e^−^. (x = 1) composite samples tend to form particles with size of about few nano-meter along with almost uniform but spherical shapes (Fig. 5(a)). Inside Fig. 5(a) shows the EDX and it confirmed that all required elements are present in the sample. Similarly, Fig. 5(b) shows TEM image, where most of particles are about 5 nm with few particles about 10 nm. The Fig. 5(c) shows HR-TEM of synthesized sample where inset Fig. 5(c) clearly shows carbon graphitized ring. The rGO formation in system can effectively avoid agglomeration and control the size of the C12A7:e^−^ nanoparticles with synergistic effect, making stable doping of Fe on reduction of C12A7 to C12A7:e^−^. Hence we first time successfully synthesized nanosize Fe-doped C12A7:e^−^ composite. For further application point of view, the conductivity and BET based surface area of the Fe-doped C12A7:e^−^ composite were studied.

### Electrical properties and BET measurements

**Figure 6:**
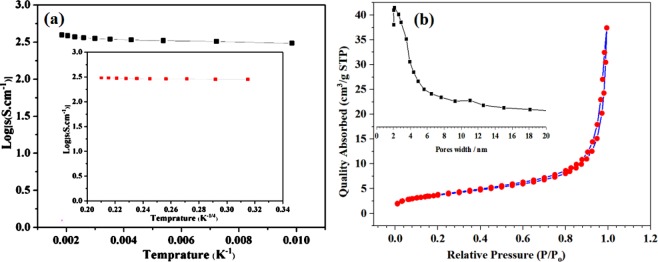
(**a**) Conductivity measurements, and (**b**) BET surface area of C_12_A_7−x_Fe_x_:e^−^, where doping level, x = 1.

The measured electrical conductivity of the Fe-doped C12A7:e^−^ composite, with doping level, x = 1, was 249 S.cm^−1^ (Fig. 6(a)). Hence, we successfully synthesized the nanosize Fe-doped C12A7:e^−^ composite with stable phases and with high conductivity. This was the first time synthesis of Fe-doped C12A7:e^−^ composites, and will further enhanced electrocatalyst properties in the fuel cell, because of its low work-function and moderate optoelectrical properties along with its abundant nature. Similarly, the estimated BET specific surface area of the Fe-doped C12A7:e^−^ composite was about 149 m^2^ g^−1^, higher than the un-doped C12A7:e^−^ (20 m^2^ g^−1^) (Fig. 6(b))^7^. Another important parameter is the pore size estimation, which was estimated using Barrett-Joyner-Halenda (BJH) method from the adsorption branches for the synthesized Fe-doped C12A7:e^−^ composites. Inside Fig. 6(b) discloses almost single sharp peak at ~3 nm, proposing that the sample has almost homogeneous pore size distribution. After examining N_2_ adsorption desorption isotherm its well matched with type IV isotherm^26^. The highest surface area maybe because of the rGO, which not only to enhance the surface area, but also did not allow the particles to agglomerate, was also confirmed by the TEM images. Next, for further confirmation of the Fe-doped C12A7:e^−^ composites electrocatalytic properties, we also investigated the XPS analysis.

### X-ray photoelectron spectroscopy (XPS)

**Figure 7:**
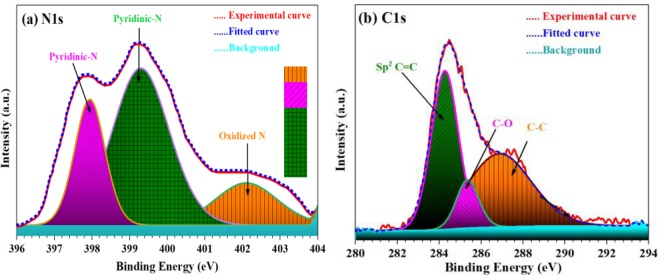
XPS spectra of Fe-doped C12A7:e^−^ composite (x = 1), (**a**) N1s, and (**b**) C1s.

To further improve our understanding of synthesized materials and its effect on the final properties of Fe-doped C12A7:e^−^ composite, XPS measurements were carried out. The XPS results shows that the pyridinic-nitrogen species (N bonded to two carbon atoms) exhibit an N1s binding energy peak at ~398.1 eV, and ~399.5 eV^27^, along with oxidized-N peak at ~402.2 eV, as shown in Fig. 7(a). Similarly, the C1s peak^28^ showed a sharp peak at 284.4 eV, which correspond to non-oxygenated rings and hence it refers to aromatic C-C (47%) bonds of sp^2^-carbon atoms in a conjugated honeycomb lattice (Fig. 7(b)). Based on this result, it can be stated that the highest peak ratio of the C-C (47%) bond is due to the formation of the C-C skeleton by the reduction process of the oxygen-containing species. Similarly, oxygenated rings peaks at 285.40 eV, and 287.921 eV could be attributed to the C in C-O (15%), and carbonyl (C-C, 38%), respectively. It can be concluded that the observations described prove that the formation of the rGO^29^.

### Electrochemical analysis

**Figure 8:**
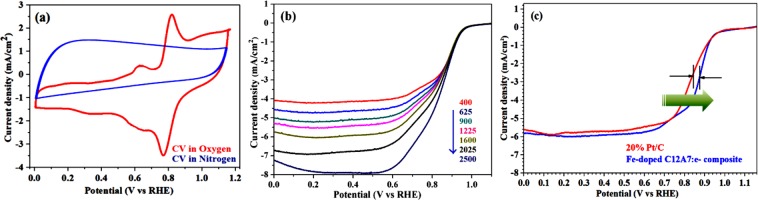
(**a**) CVs curves with a scan rate 50 mV·s^−1^ under O_2_ saturated 0.1 M KOH solution, (**b**) LSV/ curves with a scan rate 10 mV/s, **(c)** ORR polarization curves of Fe-doped C12A7:e^−^ composite, and Pt/C, in O_2_-saturated 0.1 M KOH, with 1600 rmp, and 10 mV/s scan rate.

In our proposed schemed first we doped Fe in the C12A7:e^−^, followed by coating with the rGO under the N_2_ flow^1,5,7^. The elemental chemical compositions distribution on the surface of prepared mesoporous Fe-doped C12A7:e^−^ composite robustly manipulate its electrocatalytic activity. In electrocatalytic measurements, no additional carbon was added to the Fe-doped C12A7:e^−^ composite, to augment the conductivity of the as synthesized product. The catalysts activity will be discuss in the form of onset/half-wave potential, current density, and robustness as electrode materials. This highly conductive Fe-doped C12A7:e^−^ composite, with high electron concentration, and low work-function, can easily transfer electron from C12A7:e^−^ to coated rGO and hence boost the electrochemical reaction. The onset potential measured from the CVs curves based on the RDE experimental calculations. The CVs curves of Fe-doped C12A7:e^−^ composite in O_2_ and N_2_ saturated 0.1 M KOH are given in Fig. 8(a), where a clear cathodic reduction current obtained in the O_2_ but it is not seen in N_2_ saturated 0.1 M KOH solution, it means only active as anode material in O_2_ saturated 0.1 MKOH solution. So, to obtain the supplementary approach into these positive aspects, we measured the LSV analysis. From the LSV curves half wave potential, along with the current density of Fe-doped C12A7:e^−^ composite compared to the standard Pt/C (20%). The Fe-doped C12A7:e^−^ composite ORR polarization curves (LSV) at different rotation speeds from 400–2500 rpm range, were measured for the scrutinizing catalytic routes of the synthesized Fe-doped C12A7:e^−^ composite (Fig. 8(b)).

In general the current density curves, diffusion current density becomes improved with increasing rotation rate^1^. The same fact is also observed for Fe-doped C12A7:e^−^ composite for ORR with increased rotation rate, and have adequately demonstrated high intrinsic activity as the ORR electrocatalysts. It is confirmed that future of alkaline fuel cell is highly reliable on the ORR electrocatalyst development with lofty half wave potential and the current density. Figure 8(c) shows the RDE polarization curves of the Fe-doped C12A7:e^−^ composite (doping level of x = 1), and benchmark 20% Pt/C at 1600 rpm. For Fe-doped C12A7:e^−^ composite, 0.89 V (vs. RHE) half wave potential and ~5.9 mA.cm^−2^ current density were superior than the 20% Pt/C (5.65 mA.cm^−2^, and 0.84 V), which shows the elevated electrocatalytic activity of the Fe-doped C12A7:e^−^ for ORR in fuel cell.

**Figure 9:**
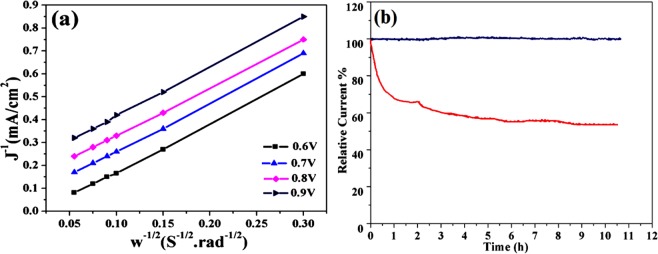
(**a**) At different potentials K-L plots of Fe-doped C12A7:e^−^ composite (doping level, x = 1), and (**b**) Chronoamperometry curves of 20% Pt/C and synthesized electrocatalysts measured at 1600rpm.

**Figure 10:**
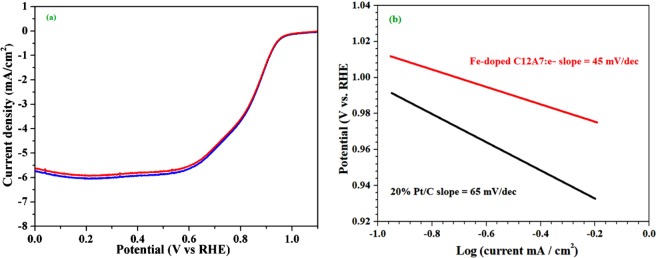
(**a**) LSV curves of the Fe-doped C12A7:e^−^ composite in O_2_-saturated 0.1 M KOH without (blue curve) and with (red curve) 1 M MeOH, (**b**) Tafel plots calculated from the RDE polarization curves.

Another important factor regarding to the application as electrocatalyst electrode material is number of electrons transfer during the ORR process^15^. Fig. 9(a) demonstrate the Koutecky-Levich (K-L) plots has good linearity and parallelism, showed the number of electron transfer in the ORR, were in 3.985 to 3.995 range for potential 0.4 to 0.8 V, which illustrate that Fe-doped C12A7:e^−^ composite followed the four-electron route^30–32^. Keeping in mind its high electrocatalytic activity, it’s expected that the Fe-doped C12A7:e^−^ composite can be used as alternative cathode electrocatalyst for the ORR applications in the place of the benchmark 20% Pt/C and grasp pledge to decrease the fuel cells expenditure. Since, commercializing for the fuel cell, catalysts should also have good stability and resistance towards the CO-poisoning and methanol crossover. Till date reported metal based electrocatalysts have low stability and immunity for methanol crossover and the CO-poisoning, which have been considered main obstacles hindering their application in the fuel cells. The long-time durability of the Fe-doped C12A7:e^−^ composite was further examined by the chronoamperometric response at a potential of 0.87 V vs., RHE in 0.1 M O_2_-saturated KOH solution, for 11 h (Fig. 9(b))^31,33^. After 11 h, the relative current of Fe-doped C12A7:e^−^ composite almost remains the same (black curve) but about 40% decrease was observed for benchmark 20% Pt/C (red curve)^30^. Furthermore, approximately there is no change in the LSV curve of the Fe-doped C12A7:e^−^ composite, can be seen upon addition of the methanol (Fig. 10(a))^30,34^.

**Figure 11:**
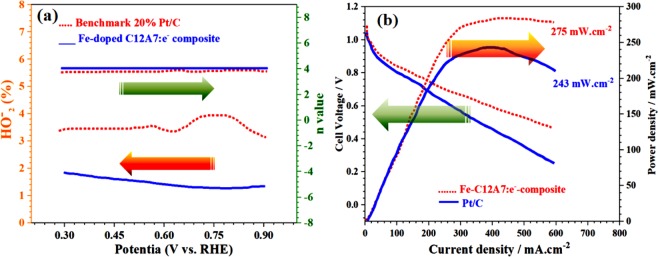
(**a**) HO_2_^−^ % yield for oxygen reduction reaction, **(b**) Cell voltage and power density versus current of Fe-doped C12A7:e^−^ composite (x = 1) catalyst as a cathode for AAEMFC.

Similarly, Fig. 10(b) shows that Fe-doped C12A7:e^−^ composite exhibits the Tafel slope of ≈45 mV/dec, smaller than the benchmark 20% Pt/C (≈65 mV.dec^-1^), on behalf of it as a exceedingly active catalyst in the fuel cell. Hence, this behavior shows that the Fe-doped C12A7:e^−^ composite electrocatalyst can also have potential application in the methanol fuel cells^30,35^. The HO_2_^−^ % yield for the ORR measured by the RRDE method shows that the Fe-doped C12A7:e^−^ composite sample HO_2_^−^ % yield is less than 2.4 and also less than benchmark 20% Pt/C, which is very useful for the fuel cell application (Fig. 11(a))^16^.

## Alkaline Anion Exchange Membrane Fuel Cells (AAEMFC) Power Density

Finally the most important characterization of the Fe-doped C12A7:e^−^ composite catalyst was tested as cathode material in a real fuel cell, and its performance in real system was assessed by membrane electrode assembly (MEA) in Alkaline Anion Membrane Fuel Cell (AAEMFC). Polarization and power density curves of H_2_/O_2_ AAEMFC with Fe-doped C12A7:e^−^ composite and benchmark 20% Pt/C are shown in Fig. 11(b). Fuel cell working was tested at operation temperature of 80 °C, H_2_ and O_2_ flow rate was set as 200 cc min^−1^, relative humidity of anode and cathode was ~100%; and the used membrane was A201 (Tokuyama)^16,36^. The measured open circuit voltages of Pt/C and Fe-doped C12A7:e^−^ composite-based AEMFCs were 1.02 and 1.03 V, respectively, which are in good agreement with trend of onset potential in the LSV. The maximum power densities can reach up to 243 mW/cm^2^ and 275 mW/cm^2^ for benchmark 20% Pt/C and Fe-doped C12A7:e^−^ composite, respectively^16,36^. Hence, the Fe-doped C12A7:e^−^ composite will be the best to use as a electrode material in energy producing and saving devices. Hence, our investigation for “Fe” doping on the electrical properties of the C12A7:e^−^ show that above results manifest the feasibility of this sol-gel method for the cation doping, for further boosting the electrocatalytic applications. It shows that the Fe-doped C12A7:e^−^ composite is very active in the ORR and exhibits higher electrocatalytic activity, which could be demonstrated from its superior current density and more positive half-wave potential. Finally, presence of the C12A7:e^−^ inside the rGO, results in the straightforward transfer of an electron from core metal C12A7:e^−^ to rGO^1^. Four-electron pathway, high catalytic activity, and durability of the rGO coated Fe-doped C12A7:e^−^ composite are superior than benchmark 20% Pt/C.

Promising electrocatalytic performance of the rGO coated Fe-doped C12A7:e^−^ composite may be translated to a wide range of applications. Atomic-scale ORR mechanism is still in early levels due to complex kinetics but we can conclude that the synthesized nanosize rGO coated Fe-doped C12A7:e^−^ composites will further boost the electrochemical properties as electrode material with high stability. This concept paves the way for a new class of hybrid electrocatalysts, where activity and stability of the electrocatalysts are addressed.

## Conclusion

**Scheme 1:**
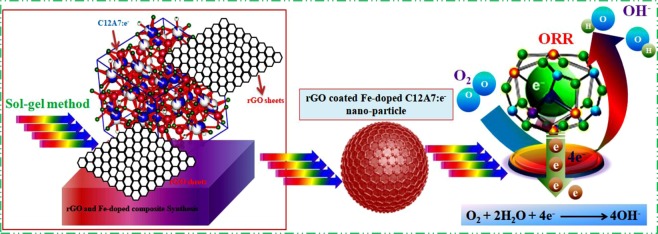
Schematic presentation of ORR on the envisage microstructure of rGO coated Fe-doped C12A7:e^−^.

In summary, this study based on our designed facile synthesis strategy for Fe-doped C12A7:e^−^ composite, effectively boost is observed in electrical properties by doping “Fe” on “Al” vacancy, at 1500 °C for 1 h, which could be a flexible podium for developing mesoporous Fe-doped C12A7:e^−^ composite. The maximum achieved conductivity was 249 S·cm^-1^, with doping level as x = 1. Fuel cells are observed as the feasible maneuver for long-term electromobility, however the PGMs requisites for catalyzing ORR is obstacle. The Fe-doped C12A7:e^−^ composite was disclosed to accomplish the supplies of high selectivity. Current density and half wave potential of the Fe-doped C12A7:e^−^ composite (doping level, x = 1), were ~5.9 mA/cm^2^ and 0.89 V (RHE), higher than that of commercially used benchmark 20% Pt/C (5.65 mA/cm^2^, and 0.84 V). Catalytic study exposed the mesoporous Fe-doped C12A7:e^−^ composite will be implement as a first-rate catalyst even exclusive of extra carbon black to further enhance conductivity. This newly synthesized, Fe-doped C12A7:e^−^ composite catalysts along extra edge of cost-effective and its profuse availability, facilitate ORR with high current density via four-electron pathway surpassing corrosive hydrogen peroxide formation. Hence, the developed protocol for active materials can be extended to the other hybrid range in electrochemical energy storage and electroatalyst sensing applications (Scheme 1).
